# The Role of mTOR Inhibitors after Liver Transplantation for Hepatocellular Carcinoma

**DOI:** 10.3390/curroncol30060421

**Published:** 2023-06-09

**Authors:** Letizia Todeschini, Luca Cristin, Alessandro Martinino, Amelia Mattia, Salvatore Agnes, Francesco Giovinazzo

**Affiliations:** 1 Faculty of Medicine and Surgery, University of Verona, 37134 Verona, Italy; 2 Department of Surgery, University of Illinois Chicago, Chicago, IL 60607, USA; 3 General Surgery and Liver Transplantation Unit, Fondazione Policlinico Universitario Agostino Gemelli IRCCS, 00168 Rome, Italy

**Keywords:** hepatocellular carcinoma, HCC, immunosuppression, liver transplantation, mTOR, mTOR inhibitors

## Abstract

Liver transplantation is a treatment option for nonresectable patients with early-stage HCC, with more significant advantages when Milan criteria are fulfilled. An immunosuppressive regimen is required to reduce the risk of graft rejection after transplantation, and CNIs represent the drugs of choice in this setting. However, their inhibitory effect on T-cell activity accounts for a higher risk of tumour regrowth. mTOR inhibitors (mTORi) have been introduced as an alternative immunosuppressive approach to conventional CNI-based regimens to address both immunosuppression and cancer control. The PI3K-AKT-mTOR signalling pathway regulates protein translation, cell growth, and metabolism, and the pathway is frequently deregulated in human tumours. Several studies have suggested the role of mTORi in reducing HCC progression after LT, accounting for a lower recurrence rate. Furthermore, mTOR immunosuppression controls the renal damage associated with CNI exposure. Conversion to mTOR inhibitors is associated with stabilizing and recovering renal dysfunction, suggesting an essential renoprotective effect. Limitations in this therapeutic approach are related to their negative impact on lipid and glucose metabolism as well as on proteinuria development and wound healing. This review aims to summarize the roles of mTORi in managing patients with HCC undergoing LT. Strategies to overcome common adverse effects are also proposed.

## 1. Introduction

Hepatocellular carcinoma (HCC) is the most common malignancy of the liver and the third cause of cancer-related mortality [1]. In 2020, the WHO reported 902,000 new diagnoses of primary liver tumours and 830,000 deaths, and these data are expected to rise in the future [1]. HBV or HCV infections are still considered the most prominent risk factor in developing HCC [2]. In underdeveloped areas, higher exposure to these infections and lower access to treatment account for the higher incidence of HCC. However, in the last three decades, a rising trend in HCC incidence has been seen in Northern America and Europe due to the increasing prevalence of NAFLD and metabolic syndrome [2,3]. Despite the progress in managing liver malignancies, the prognosis of hepatocellular carcinoma is still poor due to the tumour’s aggressiveness and the morbidity associated with the treatment. Liver transplantation (LT) is a treatment option for HCC. Indeed, the number of patients transplanted for HCC is increasing, with HCC representing 15–50% of all indications for LT performed in most centers [4,5,6,7]. However, tumour recurrence and other adverse effects of the conventional calcineurin inhibitors (CNIs)-based immunosuppressive therapy is a cause of concern. As a result, inhibitors of the mammalian target of rapamycin (mTORi) are being explored as a possible alternative through their antiproliferative and immunosuppressive action. Among the several pathways implicated in the oncogenic process, PI3K/AKT/mTOR is particularly relevant due to its constitutive activation in many patients with HCC and its association with the more severe forms of the disease [8]. The PI3K-AKT-mTOR pathway regulates protein translation, cell growth, and metabolism [9,10,11]. Evidence suggests that the mTOR pathway is involved in malignant progression and resistance to treatment through the over-activation of several mechanisms. While inhibition of the mTOR pathway demonstrates apparent effects on tumoral growth, an inhibitory impact on innate and adaptive immunity is also achieved. The mTOR pathway signalling regulates the activity of the professional antigen-presenting cells and the function and differentiation of T-cell subpopulations, accounting for its immunomodulating effect [12,13]. As a result, targeting the mTOR pathway has emerged as a promising therapeutic strategy in HCC patients’ post-liver transplantation.

This review aims to highlight the role of the inhibitors of the mammalian target of rapamycin (mTORi) in the management of patients with a diagnosis of HCC after undergoing LT (Table 1). In addition, the article provides some insight into the mTOR pathway and its clinical impact on the pathogenesis of HCC. 

## 2. Akt-mTOR Signaling Pathway

mTOR is a serine-threonine kinase of the PI3K superfamily that is found in cells as a component for mTOR complex 1 (mTORC1) or mTOR complex 2 (mTORC2) [32]. These complexes exert similar roles in cell growth, proliferation, lipogenesis, autophagy, survival, and angiogenesis [33]. Several oncogenic factors, such as platelet-derived growth factor (PDGFR), epidermal growth factor (EGFR), and mesenchymal-epithelial transition factor (METF), can activate PI3K, an upstream regulator of AKT-mTOR [34]. PI3K transduces the signals into intracellular messages by phosphorylating the 3′-OH position of the inositol ring of the second messenger phosphatidylinositol bisphosphate (PIP2) [35]. Afterwards, phosphatidylinositol triphosphate (PIP3) recruits and activates the phosphatidylinositol-dependent kinase 1 (PDK1) that phosphorylates the serine-threonine protein kinase AKT [also known as protein kinase B (PKB)] [36]. Phosphorylation of AKT is also achieved through mTORC2 activity, which is promoted by PI3K through a still poorly understood mechanism [32,37]. AKTs are serine-threonine kinases and comprise three protein isoforms (AKT1, AKT2, and AKT3) acting on cellular survival, proliferation, growth, and metabolism. AKT phosphorylates the tuberous sclerosis complex (TSC1/TSC2) and turns off its inhibitory effect on mTOR activator protein Rheb, resulting in the mTORC1 activation [38]. mTORC1 regulates cell growth and metabolism mainly by inhibiting catabolism and activating anabolism [9,10]. This complex represses autophagy through the inactivation of unc-51-like autophagy activating kinase (ULK1) and transcription factor EB (TFEB) [39,40]. Moreover, activating the mTOR pathway enhances the activity of HIF1α/VEGF, causing neovascularization and angiogenesis and the proliferation of vascular endothelial cells and some other cell types [11].

### PI3K/mTOR Pathway in HCC

**Figure 1:**
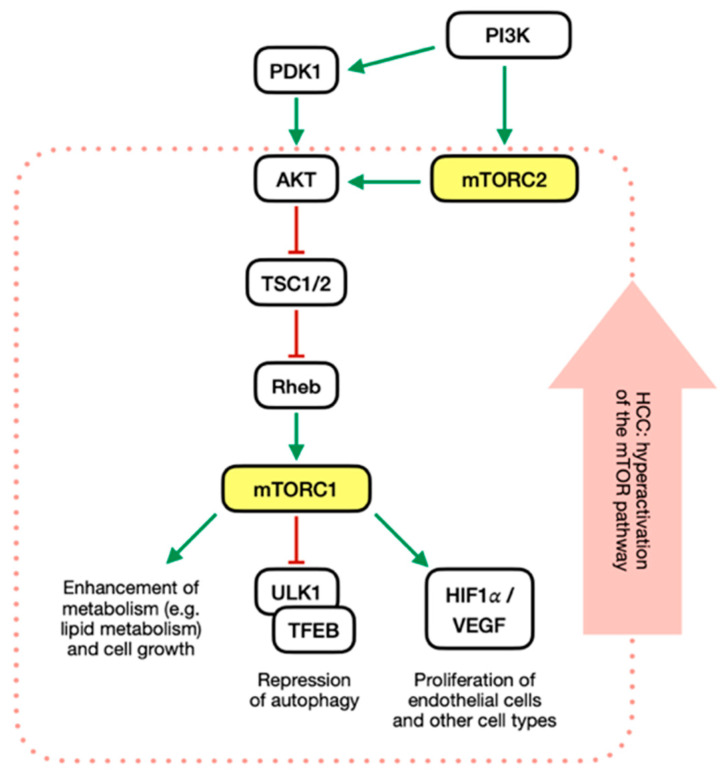
The mTOR pathway in HCC. PI3K activation following growth factors stimulation promotes PDK1 and mTORC2 activity, with an overall stimulating effect on AKT activity. AKT controls mTORC1 signalling by inactivating the inhibiting effect of the TSC1/TSC2 complex toward the mTORC1 activator Rheb. mTORC1 activation enhances cellular metabolism, represses autophagy by inhibiting ULK1 and TFEB, and promotes cellular and vascular proliferation through the HIF1𝛼/VEGF pathway. In HCC, mTORC1 and mTORC2 are upregulated, resulting in uncontrolled cell growth and the promotion of carcinogenesis.

Figure 1 Dysregulation of the mTOR pathway is a critical step in hepatocellular carcinogenesis [41,42,43]. Evidence shows its activation in HCC, hepatoblastoma, and cholangiocarcinoma [44]. Almost half of HCC patients have an upregulation of mTORC1 and mTORC2 pathways. Activation of these pathways is also associated with earlier tumour recurrence, less differentiated forms, worse prognosis, and earlier recurrence, independent of the etiology of liver cancer [45,46,47]. Hyperactivation of the mTOR signalling results in abnormal lipid metabolism and inhibition of autophagy [48,49]. However, mTOR deregulation is linked to liver cancer, also independent from liver steatosis [43]. Indeed, mice with constitutive mTORC1 activation in the liver spontaneously developed lower-grade tumours and hepatocellular carcinoma [43]. This effect was independent of liver steatosis: constitutive mTOR activation in mice caused spontaneous hepatocyte cell death, inflammation, cellular regeneration, and DNA damage that led to HCC.

The mTOR pathway expression is non-homogeneous across the tumour mass. Given the centrifugal expansion of tumour tissue, mTOR expression is expected to be more intense at the edge of cancer, where the proliferation is more significant [50]. Interestingly, the probability of HCC recurrence after LT positively correlates with the mTOR pathway activity rate within the tumour rather than the peritumoral area. The local inflammatory process commonly present at the edge of cancer can explain the effect, which may have a confounding inhibitory effect on the mTOR pathway expression in this area. Consequently, assessing the mTOR pathway expression within the mass may better reflect the proliferation rate of the tumour and, consequently, its tendency to recur [50].

## 3. Inhibitors of Akt-mTOR Signaling Pathway

Reduced activity of the Akt/mTOR pathway can be achieved with mTOR inhibitors. The most used ones are rapamycin (Sirolimus) and its derivatives (Temsirolimus, Everolimus, and Ridaforolimus). This review will focus on Sirolimus and Everolimus, since they are the two compounds mainly used in the setting of solid organ transplantation.

### 3.1. Sirolimus

Rapamycin, or sirolimus (SRL), is a first-generation non-selective allosteric inhibitor of mTORC1 and mTORC2 first isolated from the soil bacterium *Streptomyces hygroscopicus*. Considering its established role in allograft rejection suppression [51], this drug was approved in 1999 by the FDA for treating kidney transplant recipients in addition to a complete immunosuppressive regimen of cyclosporine and prednisone [52]. As of today, SRL has not been approved for treating LT patients. However, thanks to its immunosuppressive effect, it can be used with an off-label indication in mTORi-based regimens. SRL has a long terminal half-life (48–72 h), and, indeed, a daily administration is generally sufficient [53]. SRL is poorly water soluble, has poor absorption, and has a wide distribution that accounts for a low bioavailability (approximately 14–18%). Extensive interaction with other drugs has to be noted, given the role of CYP3A4 in the SRL metabolism [54].

### 3.2. Everolimus

Everolimus (EVR) is a rapalog with similar immunosuppressive, anti-angiogenic, and antiproliferative effects as sirolimus. However, it has better efficacy and activity, which optimizes clinical use. EVR has been approved for the prevention of graft rejection after 2012. Everolimus complexes with cytoplasmic protein FKBP-12 and inhibits mTOR, suppressing p70 S6 kinase phosphorylation. EVR has comparable low absorption, bioavailability (20%), and interaction issues due to CYP3A4-dependent metabolism. Terminal half-life is slightly shorter (26–30 h), but even in this case, a single oral dose is sufficient to maintain adequate blood concentrations [53].

## 4. mTORi and HCC Recurrence

Recurrence is one of the significant issues in HCC management, occurring in approximately 16% of patients undergoing liver transplantation. Overall, survival after recurrence is generally 1 year [55]. While tumour characteristics are the main predictors of HCC recurrence, immunosuppressive regimens have also been pointed out to play an important role [56]. In post-LT patients, CNI-based regimens exhibit a negative predictive effect on recurrence and malignancy progression in a dose-dependent fashion [29,56,57]. This effect is due to CNIs’ ability to switch off the immune system’s response, promoting cancer cell survival [58]. Ultimately, traditional immunosuppressors create a permissive environment for tumoral cell growth, accounting for a higher risk of recurrence in LT recipients [56]. Similar effects on the immune microenvironment have been described for anti-lymphocyte antibodies, another drug option available post-LT induction [59].

Moreover, alteration of the host’s immune response correlates with an acceleration in tumoral growth rate [60]. The tumoral growth may be related to the ability of CNIs to induce overexpression of TGF-β1, thus promoting proliferation and aggressiveness [61]. Acknowledging the role of the mTOR pathway in the proliferation and survival of tumoral cells, rapamycin and its derivatives can be considered a possible solution to addressing this issue. An antiproliferative effect is consequent to inhibiting proliferative pathways usually induced by mTOR complexes. Moreover, an anti-angiogenic effect results from reduced activity of HIF1α/VEGF following inhibition of the mTOR pathway. Along with these two properties, the ability to preserve an adequate immune response against tumour cells makes it a valid option in both the prevention and the treatment of HCC recurrence.

### 4.1. Prevention of HCC Recurrence

The choice of a mTORi-based regimen over other immunosuppressive options correlates with lower HCC recurrence probability. mTOR inhibitors exhibit an independent positive predictive value on recurrence rate, which can be demonstrated when administered alone or in combination with reduced CNIs [62]. Reducing CNIs rather than total withdrawal is justified because its correlation with HCC recurrence is dose-dependent and stronger when CNIs are higher [63].

#### 4.1.1. Sirolimus

A revision of several retrospective studies conducted between 1999 and 2022 identified the advantages of a sirolimus-based regimen over other immunosuppressive options in LT recipients [64]. A lower recurrence rate was found when sirolimus was the drug of choice, particularly in patients within the Milan criteria [64]. A positive impact on recurrence-free survival (RFS) has also been demonstrated in patients treated with sirolimus. In the SiLVER study, randomization to sirolimus or a mTORi-free regimen showed a higher RFS rate when sirolimus was included in the treatment (70.2% vs. 64.5%, *p* = 0.28), with an average gain of RFS of 6.4 months compared to controls [21]. Only at 1 and 3 years after LT a statistically significant difference was found in RFS between the two groups (92.5% vs. 85.2%, *p* < 0.0125; 80.6% vs. 72.3%, *p* < 0.0499), while later on, this advantage no longer existed [21]. A 13% to 15% higher RFS was found when sirolimus was administered in monotherapy rather than combined with other immunosuppression. Still, the low proportion of patients did not allow the study to reach significant conclusions [21]. When a risk stratification based on the Milan criteria was made, a higher RFS rate at 4 years was found in low-risk patients treated with sirolimus. However, a benefit was not achieved if the risk was high, suggesting that sirolimus may have a more evident advantage in the early stages of the disease [21].

Moreover, younger recipients (≤60 years) have a more significant advantage [21]. It is believed that sirolimus advantages are correlated to a later tumour redevelopment and more prolonged survival after recurrence [25]. This effect was later demonstrated even in recipients exceeding the Milan criteria, justifying its utilization in more advanced types of HCC [14].

#### 4.1.2. Everolimus

A beneficial effect on RFS and recurrence rate was also found for everolimus-based regimens. Evaluation of RFS in patients treated with an everolimus-based regimen showed a higher rate at 1 year (RR 1.09, 95% CI 1.01–1.18) and 3 years (RR 1.1, 95% CI 1.01–1.21) compared to CNI-treated patients. Moreover, overall recurrence rate was lower in the mTORi group (RR 0.67, 95% CI 0.56–0.82) [65]. A higher recurrence rate was found in patients exceeding the Milan criteria in both the groups examined [66]. This result is consistent with the SiLVER study findings for sirolimus-treated patients [21]. According to a retrospective analysis by Sapisochin et al., an early start of everolimus administration is advised. The authors found that early initiation of an everolimus-facilitated tacrolimus (TAC) reduction lowers the rate of HCC recurrence compared to tacrolimus control (3.6% vs. 11.5%, *p* = 0.136) at 5 years after LT [30]. Moreover, analysis of patients who received an everolimus-based immunosuppressive regimen showed a correlation between drug blood concentration and recurrence rate, with lower HCC recurrence rates in patients with mean trough levels of everolimus >6 ng/mL [67].

### 4.2. Treatment of HCC Recurrence

Recurrence of HCC is associated with poor prognosis in affected patients, with a median survival expectation of less than 1 year [68]. After liver transplantation, HCC recurrence is mostly extrahepatic only (50–60%), arising in the lungs or the bone, but sometimes it can simultaneously involve intrahepatic sites (30–40%) [69,70]. Less frequently, an intrahepatic-only recurrence can occur (15–40%) [69,70]. Management of local recurrence is based on resection with curative purpose since this approach has shown to be more beneficial than the implementation of palliative care or best supportive care [71]. However, resection is not an effective treatment option for most patients since recurrence occurs more frequently in extrahepatic sites. In the presence of a disseminated HCC recurrence, systemic therapy with sorafenib (SOR) represents the strategy of choice [72]. Sorafenib is a multikinase inhibitor of the vascular endothelial growth factor receptor (VEGFR) family and platelet-derived growth factor receptor (PDGF) family [73,74]. Sorafenib administration benefits the patient’s prognosis, correlating with a pooled 1-year survival of 63% and a median survival of 12 months [75]. This multikinase inhibitor can be administered in monotherapy or combined with immunosuppressive therapy. Combination with mTORi accounts for the stabilization of the disease and a median overall survival of 19.3 months [17]. The impact on 1-year OS is more significant when SOR + mTORi is chosen over SOR monotherapy (*p* = 0.03), suggesting a more significant role of mTORi [23]. To assess which of these two drugs has the most beneficial effect on post-recurrence survival, 232 LT recipients with HCC recurrence were evaluated [22]. They were divided into four groups according to the treatment chosen: SOR, mTORi, SOR + mTORi, and controls. While survival rates were not affected by SOR administration (*p* = 0.17), an improvement in survival was found following the administration of mTORi (*p* < 0.001) or SOR + mTORi (*p* = 0.011). In patients treated with mTORi, co-administration with SOR showed no difference in the overall post-recurrence survival period (*p* = 0.26) compared to mTORi monotherapy, indicating an absence of a synergistic or additional antitumor effect from sorafenib [22]. When everolimus is chosen for a mTORi-based regimen, a correlation between its blood concentration and the survival rate has also been seen. Notably, the mean survival time in patients treated with everolimus is longer when drug blood levels are ≥5 ng/mL both in patients treated with everolimus alone (19.9 months vs. 10.7 months; *p* = 0.021) or in combination with sorafenib (22.5 months vs. 10.7 months, *p* = 0.030) [28].

### 4.3. Prevention of De Novo Malignancy

Incidence of de novo malignancy (DNMs) after LT is reported in 3–14% of recipients, with skin cancers and hematologic malignancies being the most common types [76]. According to the ILTS-SETH consensus, immunosuppression plays a crucial role in de novo tumorigenesis while predisposing a more aggressive behavior explains the higher mortality rate registered in LT recipients [76]. Discordant conclusions were drawn by a large French national study that found no impact of initial immunosuppression on DNMs occurrence [77]. However, this may be due to the substantial heterogeneity in the immunosuppressive regimens administered to patients and the numerous scheme changes, which did not allow a correct interpretation of the available data. Interestingly, an increased risk of DNM has been demonstrated for prolonged exposure to CNI-based regimens but not for mTOR inhibitors [29]. Indeed, mTOR inhibitors correlate with a lower incidence of DNMs, an effect that can be reconducted to the antiproliferative properties of this type of drug [78]. Therefore mTOR inhibitors over CNIs are advised in patients at risk for de novo malignancy [78]. Multiple factors can contribute to increasing the risk for DNMs, including an underlying liver disease (e.g., alcoholic cirrhosis), concurrent inflammatory bowel disease, human-herpesvirus-8 positivity, or de novo development of Epstein-Barr virus DNA positivity after transplantation [78].

## 5. Impact on Overall Survival

While LT is considered the best option in treating nonresectable early-stage HCC patients, the mortality rate among recipients is still high (9%) [79]. The addition of an immunosuppressive regimen with CNIs led to a drastic reduction of the 34% mortality rate achieved with the previous treatment options, albeit with still high morbidity related to nephrotoxicity (8% at 1 year) and high recurrence risk (13% at 3 years) [80,81,82]. Regarding overall survival, a non-inferiority to CNIs has been demonstrated for both sirolimus and everolimus. Two recent meta-analyses investigating the effects of mTORi on LT recipients for HCC found an improvement in OS at 1-2-3-5 years in retrospective and cohort studies, along with an expected lower nephrotoxicity [65,83]. Similar results were found in a retrospective study comparing patients treated with sirolimus or an FK506-based protocol, which described a benefit in terms of 1- and 2-year overall survival (90.67% vs. 61.60%, 80.59% vs. 53.90%, *p* = 0.011) and mean overall survival (594 ± 35 days vs. 480 ± 42 days, *p* = 0.011) in the sirolimus group [14]. A better effect on OS can be achieved when mTOR inhibitors are used for a more extended period. In the SiLVER study, a more prolonged exposure (≥3 months) to sirolimus after LT in HCC patients reduced the hazard of death and improved OS [25]. This effect is best obtained when the tumour is more active, when AFP levels before LT are higher (≥10 ng/mL, HR: 1.84; 95% CI: 1.36–2.48; *p* < 0.001). Inclusion within the Milan criteria was another predictor of higher OS [25]. A benefit on OS in patients treated with sirolimus was also found when they exceeded the Milan criteria. Ferreiro et al. demonstrated that choosing everolimus over CNIs in treating LT recipients with a high risk of recurrence defined by exceeding the Milan criteria led to an improved survival rate at 5 years (60.2% vs. 32.3%, *p* = 0.05) [20]. Differences in OS also depend on the timing of administration of mTOR inhibitors. In a retrospective analysis evaluating LT recipients treated with everolimus-based maintenance therapy, initiation of everolimus immediately after LT ensured higher survival rates at 1 year (89%) compared to switch within 3 months (83%) or later (67%). On the other hand, no significant difference was found when everolimus was used alone or in combination with CNIs or mycophenolate, suggesting once again the independent positive value of mTOR inhibitors [26].

## 6. mTORi in Immunosuppression after LT

Given the mTOR pathway’s role in cellular survival and replication, its inhibition with rapamycin and rapalogs has been established as an immunosuppression option in patients undergoing solid organ transplantation. mTOR inhibitors reduce the risk of graft rejection, thus providing a better outcome for LT recipients. While liver transplantation has provided a new chance for selected patients with HCC, this option has been burdened by a high mortality rate, which in patients treated with azathioprine reached a value of approximately 70% at 1 year [84]. These dramatic results were mitigated by implementing a stricter patient selection and introducing a CNIs-based immunosuppressive regimen. Notably, the latter led to a drastic reduction in the mortality rate, settling at a little over 20% [84]. Cyclosporine and tacrolimus are the drugs of choice in the early treatment of LT recipients, thanks to their ability to block T-cell activation and migration [85]. Both tacrolimus and cyclosporine inhibit calcineurin, a cytoplasmic protein responsible for IL2 gene expression, and reduce the production of chemotactic factors involved in lymphocyte recruitment [85]. Utilizing CNIs gave obvious benefits in post-LT management, but their nephrotoxicity and high risk of recurrence required reduced CNI-exposure treatment [29,56,57,86]. In the last 20 years, a growing interest has been developed in mTOR inhibitors, as an immunosuppressive effect was reported in both in vitro and in vivo assessments [87]. This effect is achieved thanks to the ability of rapamycin and its derivatives to induce T-cells anergy while promoting an allograft tolerance through increasing Tregs levels to the detriment of other CD4+ cell subpopulations [13]. mTOR inhibitors also exhibit an antiproliferative effect, making it an optimal option in managing LT recipients with an HCC as an indication, particularly when the Milan criteria were fulfilled [88].

### 6.1. Immunosuppressive Regimens

Recipients receive an immunosuppressive regimen after LT to reduce allograft rejection probability. CNIs (cyclosporine and tacrolimus), glucocorticoids, and mycophenolate mofetil are the drugs of choice in the settings of initial combined therapy. When stability in liver function is obtained, monotherapy is advised, usually CNIs-based. Because of their nephrotoxic side effects, CNIs reduction or discontinuation is recommended in the long term [88]. In HCC patients, mTOR inhibitors are an appropriate option to achieve this purpose, and their beneficial effects are discussed. As of today, a conversion to mTORi-based therapy is advised within 3 months from LT, with the possibility to administer these drugs in monotherapy or in combination with reduced CNIs [88]. Care must be taken when everolimus is the drug of choice for tacrolimus reduction, as a target range of 3 ng/mL should be achieved before the reduction of the CNI [78].

Other therapeutic combinations have been explored other than mTOR inhibitors with reduced-CNIs. Co-administration of mTORi with mycophenolate (MPA) is one of the possible options, as it improves renal function and quality of life 24 months after liver transplantation compared with standard CNI with MPA immunosuppression [27].

### 6.2. Impact on Renal Function

Nephrotoxicity is a well-established adverse effect of the long-term utilization of CNIs. Endothelial dysfunction and vasoconstriction play an essential role in CNIs renal damage, which can be traced back to ROS production, RAS activation, and induction of imbalances in vasodilators and vasoconstrictors production [89]. Renal damage becomes one of the most essential complications in transplanted patients, accounting for increased morbidity and mortality [90]. Indeed, renal dysfunction is the main indication for discontinuing CNIs, and mTORi are optimal alternatives thanks to their renoprotective effect. However, given that both sirolimus and everolimus serve this scope, other studies are needed to determine which drugs are more effective in achieving a long-term renoprotective effect [91].

#### 6.2.1. Sirolimus

A significant improvement in renal function was found when SRL was administered to transplanted HCC patients who developed nephrotoxicity after a first course of CNIs. This effect was mainly present in patients that started the sirolimus regimen within 3 months from LT. In these recipients, eGFR increased from 30 mL/min to 57 mL/min [16]. Similar results may be achieved when low doses of sirolimus are administered along with reduced tacrolimus. Significantly fewer patients had a CKD grade ≥3 at 6 months when low sirolimus + low tacrolimus was chosen over a conventional tacrolimus regimen [31].

Conflicting results came from a meta-analysis conducted by Asrani et al., who found that the beneficial effect on renal function after conversion to sirolimus was non-significant [92]. However, this result may be explained by selection bias and late conversion (>6 months) to the sirolimus regimen. Evaluation of patients treated with sirolimus showed they had the most impaired renal function baseline compared to controls. Moreover, sirolimus was believed to be administered later than needed in patients with preserved renal function, promoting its degeneration in this window period [92]. When conversion from CNIs to sirolimus is needed, an early switch is recommended as it gives greater possibilities for renal function recovery. Retrospective evaluation of renal function in patients undergoing early (within 3 months) or late conversion from CNIs demonstrated significantly higher eGFR mean values at all time points in the first group [15].

#### 6.2.2. Everolimus

An everolimus-based regimen preserves and, in some cases, improves renal function [93]. In the H2304 study, a significant difference of 8.5 mL/min/1.73 m^2^ (*p* < 0.001) was found in eGFR values at any point from week 6 post-LT in patients treated with everolimus plus reduced tacrolimus over controls treated with tacrolimus alone [18]. Even when a complete conversion was chosen over a reduction of the previous CNI-based regimen, a statistically significant increase in GFR values was noted in patients treated with an everolimus [19]. When an everolimus + mycophenolate regimen was chosen for CNIs conversion, a statistically significant improvement in eGFR was achieved at both 12 (88.01 vs. 60.63 mL/min/1.73 m^2^, *p* = 0.020) and 24 (87.37 vs. 53.29 mL/min/1.73 m^2^, *p* = 0.013) months compared to the CNI arm [27]. As described for sirolimus, a better outcome in renal function can be achieved when conversion to an everolimus-based regimen occurs sooner. Indeed, in LT recipients with eGFR <60 mL/min/1.73 m^2^, a more substantial improvement in renal function at 36 months was achieved when an early conversion (within 12 months) was chosen (55% if conversion within 3 months, 39.4% if conversion at 4–12 months, 20.9% if conversion was after 12 months) [24].

### 6.3. Impact on Graft Rejection

With the introduction of immunosuppressive therapy after LT, the rate of acute cellular rejection (ACR) and chronic rejection (CR) has reduced to 15–25% and 3–17%, respectively [94]. When mTORi are used in LT recipients, no difference can be found in acute graft rejection rate compared to a CNI-based regimen (RR 1.1, 95%, CI 0.94–1.28) [65]. This non-inferiority in preventing graft rejection has been proven for both sirolimus and everolimus and can be ascribed to mTORi suppressive action on the immune system [20,30,92,95]. Mainly, rapamycin and its derivatives promote the expansion of Treg at the expense of other CD4+ subgroups by altering APCs activity and T cells polarization, thus favoring immunological tolerance against the graft [13,59,96]. APCs’ activity is also affected by the inhibition of the mTOR pathway. It has to be mentioned that CD8+ T cells are preserved, accounting for the added benefit of granting an immunological response to viral infections that may occur in this period of immunodeficiency.

While a clear benefit can be described when mTORi is used to convert from a CNIs regimen, a de novo administration has not shown to be an advantage in acute graft rejection rate compared to CNIs [97,98]. This evidence may be explained by the fact that, compared to CNIs, mTORi are not equally capable of interfering with the acute expression of inflammatory cytokines. A CNI-based induction course followed by a mTORi-based regimen is thus advisable [99]. Moreover, a reduction rather than withdrawal of CNI is recommended, as evaluation of tacrolimus elimination in LT recipients has shown a higher rejection rate at 1 year (19.9%) compared to everolimus plus reduced tacrolimus (3.7%, and even tacrolimus controls (10.7%) [18].

## 7. Tolerability and AEs

As with other drugs, mTOR inhibitors are not exempt from adverse effects, which have indeed been the leading cause of reluctance to use in LT recipients. In 2002, a black box filed by US Food and Drug Administration warned about the correlation between de novo treatment with sirolimus and an increased rate of hepatic artery thrombosis found in an ongoing phase II trial. This correlation was later refuted as no hepatic vessel thrombosis events were reported when a sirolimus-based regimen was chosen over a CNI-based one [100]. It has been demonstrated that the safety profile of mTOR inhibitors is non-inferior to other immunosuppressors, paving the way toward their safe utilization [63]. The most relevant adverse effects of mTOR inhibitors and some precautions to minimize their negative impact are presented in Table 2.

### 7.1. Dyslipidemia

Dyslipidemia is found in up to 66% of LT recipients, more commonly as hypertriglyceridemia [104]. The immunosuppressors administered in LT recipients play a crucial role in lipid dysregulation, contributing to an increase in cholesterol and triglyceride levels in a dose-dependent manner [104]. Considering mTOR role in lipid metabolism regulation, inhibition of its pathway can result in the alteration of serum lipid levels. The effect is due to inhibiting lipid uptake and storage in adipocytes, hepatic lipogenesis stimulation, and lipid clearance impairment with consequent accumulation in the bloodstream [64]. While dyslipidemia has also been observed after administering mTOR-free immunosuppressive regimens, a stronger correlation was found in patients treated with sirolimus or everolimus [105]. Rapamycin administration is associated with hypertriglyceridemia and hypercholesterolemia in 50% and 45% of the patients, and drug discontinuation improves these alterations [64]. The consensus from an Italian study group identified dyslipidemia as a dose-dependent adverse effect of everolimus-based regimens in patients undergoing LT, especially when drug levels are higher than 8 ng/mL [78]. Reduction of everolimus exposure in association with statins and other dyslipidemia drugs is advised to achieve the recommended LDL levels [78,106,107]. Due to hypercholesterolemia, higher cardiovascular risk should be expected in patients treated with mTORi. However, the rate of major cardiovascular events after everolimus administration in solid organ transplant events is comparable to or lower than with mTORi-free regimens [93].

### 7.2. Hyperglycemia

Hyperglycemia is a common complication of immunosuppressor’s administration and accounts for increased mortality when sustained in time [104]. Rapamycin-based regimens are associated with the development of post-LT diabetes mellitus, with a 3-fold increase in the risk of hyperglycemia in patients treated with everolimus (17%) compared to controls (5.8%) [101]. Hyperglycemia may be due to rapamycin’s ability to induce pancreatic β-cell apoptosis, reduce pancreatic β-cell function, enhance hepatic gluconeogenesis, reduce glucose uptake, and promote insulin resistance [108,109,110]. As seen in renal recipients developing insulin resistance after mTORi administration, lifestyle modifications with or without a pharmacological approach to diabetes mellitus should be explored [111].

### 7.3. Proteinuria

The protective effect on renal function is the main indication for using mTORi in LT recipients induced with CNIs. However, proteinuria can be a possible side effect when these drugs are administered, especially when used de novo [93]. In particular, proteinuria of >1 g/d can be found at 3 years in 3% of LT recipients treated with everolimus [78]. Yet, such levels should not arouse particular concern as they are far from nephrotic ranges and may be explained by a worse renal function baseline after CNI induction instead [78]. Other evidence indicates no differences in urinary protein levels compared to controls or a worsening of pre-existing non-nephrotic proteinuria [18,92]. On the other hand, mTORi-related proteinuria may worsen underlying renal dysfunction [78]. When the patient develops mTOR-induced proteinuria >800 mg/d, constant monitoring is advised to promptly discontinue everolimus when a nephrotic range is reached [78]. If not sufficient, ACE inhibitors (ACEi) and angiotensin receptor blockers (ARBs) can be administered to achieve a renoprotective effect.

### 7.4. Wound Healing Complications

Wound healing complications (WHCs) are among the most frequent post-surgical complications, and mTORi-based immunosuppressive regimens seem to increase this risk. This evidence is primarily valid for heart and kidney transplants but is not strong enough in liver recipients [93]. A higher rate of WHCs or dehiscence was reported in patients treated with sirolimus compared to sirolimus-free regimens (30 out of 264 patients vs. 16 out of 261 patients) in a randomized controlled trial [21]. However, it has to be noted that this result may depend on the decision to delay sirolimus introduction by 4–6 weeks to minimize the risk of WHC. Similar considerations can be made for the results of the H2304 study, in which a relative risk of WHCs was reported for therapeutic regimens based on everolimus + reduced tacrolimus (11%), tacrolimus elimination (9.6%), and tacrolimus (7.9%) being reported [18]. Even in this case, the authors acknowledged a waiting period of 1 month before tacrolimus initiation to support initial wound healing [18]. These results may suggest the importance of the delayed introduction of mTORi as a protective strategy against WHC. Other preventive actions in patients at risk for WHCs are advised. Administration of the minimum effective doses of mTOR inhibitors associated with low doses of CNIs has demonstrated a similar WHC rate as MPA + CNI-treated patients [112]. Caution should be prescribed when treating patients with obesity, and efforts should be made to manage healing complication-predisposing factors [112].

### 7.5. Hematologic Adverse Effects

Hematological adverse effects can be observed in patients treated with mTORi after solid organ transplantation. 14% of the recipients treated with mTORi develop microcytic anemia, which is thought to be related to alteration in iron metabolism and in differentiation and proliferation of erythroid progenitor cells [102,113]. Neutropenia and thrombocytopenia can also be detected in 11% and 9% of the patients, respectively [113]. Dose adjustment of mTORi to the minimum therapeutic level is advised to reverse these hematologic adverse effects, but anemia can persist even after drug discontinuation [102,114].

### 7.6. Mucosal and Integumental Adverse Effects

In patients receiving mTORi after a solid organ transplant, disorders of the oral mucosa and the teguments are considered the most frequent side effects [102]. Considering LT recipients treated with a SRL-based immunosuppressive regimen, leg edema is observed in more than half of the cases (57%) [103]. Other adverse effects include oral ulcers (24%) and acne-type dermatitis (25%) [103]. While these events usually do not influence the prognosis directly, they can have a strong psychological impact, thus limiting the patient’s compliance [115]. Management of mucosal adverse effects relies on preventive strategies (e.g., oral hygiene, avoidance of irritant food and beverages) or topical treatment of the lesion with steroids, nonsteroidal anti-inflammatory drugs, or anesthetics [116,117]. Dose adjustments or drug discontinuation may be warranted for cutaneous disorders that do not resolve spontaneously, or for mucosal lesions non-responsive to previous treatments [118].

## 8. Conclusions

mTOR inhibitors represent a clear answer to the limitations of traditional immunosuppressive options in managing LT recipients. A non-inferiority to more potent immunosuppressors in reducing graft loss risk has been proven. Indeed, they represent equally effective but safer alternatives to CNIs and other immunosuppressants. Improvement of the renal impairment caused by exposure to CNI is undoubtedly the main advantage obtained with mTORi. In addition, lower HCC recurrence and de novo malignancy rates must also be mentioned. As expected, some adverse effects have been reported, with metabolic abnormalities being the primary source of concern. However, precautions can be taken to reduce the impact of these side effects and improve tolerability. Indeed, overall survival has proven higher when mTORi are chosen in a post-LT setting. The present review aims to highlight the benefits of mTORi administration in HCC patients undergoing LT. Currently, the literature lacks studies evaluating the impact of dosing and timing of administration of mTORi on the clinical outcome.

## Figures and Tables

**Table 1:** Studies included in our review to evaluate the role of mTORi after liver transplantation for hepatocellular carcinoma.

Study, Year	Study Design	Country	Populations Studied	Results
Zhou et al., 2008 [14]	R	China	73 consecutive patients who underwent LT for HCC exceeding the Milan criteria, treated with an SRL-based regimen (*n* = 27) or an FK506-based regimen (*n* = 46)	Benefit in terms of 1-year survival (594 ± 35 days vs. 480 ± 42 days, *p* = 0.011) and RFS (519 ± 43 days vs. 477 ± 48 days, *p* = 0.234) in the SRL group
Rogers et al., 2009 [15]	R	USA	72 LT recipients converted to SRL	Significantly higher eGFR mean values at all time points when the conversion was early (within 3 months)
Vivarelli et al., 2010 [16]	R	Italy	78 LT recipients treated with SRL in a CNI-sparing regimen (*n* = 38) or in combination with CNIs (*n* = 40)	eGFR increased from 30 mL/min to 57 mL/min in patients that started SRL within 3 months from LT
Gomez-Martin et al., 2011 [17]	RUC	Spain	31 patients who suffered from HCC recurrence after LT converted to mTORi-based immunosuppression plus systemic SOR	Stabilization of the disease and a median overall survival of 19.3 months are achieved using combination therapy with mTORi
De Simone et al., 2012 [18]	RCT	Multicenter worldwide	242 de novo LT patients randomized to EVR with TAC elimination (*n* = 231), EVR + reduced TAC (*n* = 245), or standard TAC (*n* = 243)	Significant difference of 8.5 mL/min/1.73 m^2^ (*p* < 0.001) in eGFR values at any point from week 6 post-LT in patients treated with EVR + low TAC Higher rejection rate at 1 year after LT when TAC is eliminated (19.9%), compared to EVR + reduced TAC (3.7%) and even TAC controls (10.7%)
Fischer et al., 2012 [19]	RCT	Multicenter worldwide	203 LT recipients initially treated with basiliximab/CNIs randomized to an EVR-based regimen (*n*= 101) or CNI continuation (*n* = 102)	Statistically significant increase in GFR values in the EVR group
Ferreiro et al., 2014 [20]	CS	Spain	52 LT recipients with a high risk of post-transplant recurrence receiving EVR (*n* = 21) or CNIs (*n* = 31) after a first course of CNIs-based immunosuppression	Higher survival rate at 5 years (60.2% vs. 32.3%, *p* = 0.05) in the EVR group
Geissler et al., 2016 [21]	RCT	Multicenter worldwide	525 LT recipients with HCC initially receiving mTORi–free immunosuppression randomized to mTORi–free regimen (*n* = 264) or an SRL-based regimen (*n* = 261)	Higher RFS rate in the SRL group (70.2% vs. 64.5%, *p* = 0.28), with a statistically significant difference only at 1 and 3 years after LT (92.5% vs. 85.2%, *p* < 0.0125; 80.6% vs. 72.3%, *p* < 0.0499) Average gain of RFS of 6.4 months in the SRL group In the SRL group: 13% to 15% higher RFS when SRL was administered in monotherapy; higher RFS rate at 4 years in low-risk patients; more significant advantage for younger recipients (≤60 years)
Jung et al., 2018 [22]	R	Korea	232 patients who suffered from HCC recurrence after LT treated with SOR (*n* = 54), mTORi (*n* = 16), SOR + mTORi (*n* = 23), or none of them (*n* = 139)	Survival rates are not affected by SOR administration (*p* = 0.17) but improve following the administration of mTORi (*p* < 0.001) or SOR + mTORi (*p* = 0.011) No difference in the post-recurrence OS period between combination or monotherapy in mTORi-based regimens (*p* = 0.26)
Invernizzi et al., 2020 [23]	R	Italy	50 patients with HCC-recurrence after LT treated with SOR	Impact on 1-year OS is more significant with a SOR + mTORi regimen (*p* = 0.03)
Saliba et al., 2020 [24]	OS	France	LT recipients receiving EVR	Better improvement of renal function at 36 months in LT recipients with eGFR <60 mL/min/1.73 m^2^ undergoing CNIs conversion within 12 months (55% if within 3 months, 39.4% if at 4–12 months, 20.9% if after 12 months)
Schnitzbauer et al., 2020 [25]	R	Multicenter worldwide	508 patients of the intention-to-treat analysis from the SiLVER study [21]	Later tumour redevelopment and more prolonged survival after recurrence in the SRL group Prolonged SRL exposure after LT (≥3 months), higher AFP levels before LT (≥10 ng/mL, HR: 1.84; 95% CI: 1.36–2.48; *p* < 0.001), and inclusion within Milan criteria are predictors of higher OS and reduced danger of death
Tejedor-Tejada et al., 2020 [26]	R	Spain	111 LT recipients treated with a mTORi-based immunosuppression	Higher survival rates at 1 year when EVR is initiated immediately after LT (89%) compared to switch within 3 months (83%) or later (67%). No significant difference was found when EVR was used alone or in combination with CNIs or MPA
Kadry et al., 2021 [27]	RCT	USA	24 LT recipients randomized to a EVR + MPA-based regimen (*n* = 12) or CNI + MPA-based regimen (*n* = 12)	Improved renal function at 12 (88.01 vs. 60.63 mL/min/1.73 m^2^, *p* = 0.020) and 24 (87.37 vs. 53.29 mL/min/1.73 m^2^, *p* = 0.013) months after LT in the EVR + MPA group
Nitta et al., 2021 [28]	RCS	France	308 consecutive patients who underwent LT for HCC	Longer mean survival time when EVR ≥5 ng/mL in patients treated with EVR alone (19.9 months vs. 10.7 months; *p* = 0.021) or in combination with SOR (22.5 months vs. 10.7 months, *p* = 0.030)
Rodríguez-Perálvarez et al., 2022 [29]	qC	Spain	425 patients who developed malignancy after LT and 425 matching controls, selected among an eligible cohort population comprising 2495 LT patients who received TAC-based immunosuppression	Increased risk of DNM has been demonstrated for prolonged exposure to CNI-based regimens but not for mTOR inhibitors
Sapisochin et al., 2022 [30]	P, R	Multicenter worldwide	86 LT recipients treated with EVR + reduced TAC (*n* = 41) or TAC (*n* = 45)	Lower rate of HCC recurrence at 5 years after LT when an EVR-facilitated TAC reduction is initiated early (3.6% vs. 11.5%, *p* = 0.136) Lower recurrence rates when mean trough levels of EVR >6 ng/mL
Mulder et al., 2023 [31]	RCT	Multicenter worldwide	196 LT recipients randomized to SRL + low TAC (*n* = 98) or TAC (*n* = 98)	Significantly fewer patients had a CKD grade ≥3 at 6 months in the low SRL + low TAC group

RCT: randomized controlled trial; R: retrospective study; P: prospective study; qC: quasi-cohort study; RUC: uncontrolled retrospective study; CS: case series; mTORi: mammalian target of rapamycin inhibitor; SRL: sirolimus; EVR: Everolimus; CNI: calcineurin-inhibitor; TAC: tacrolimus; SOR: sorafenib; HCC: hepatocellular carcinoma.

**Table 2:** Main adverse effects associated with mTORi-based immunosuppressive regimens after LT and their management.

Adverse Effects (AEs)	Rate of AEs in Patients Treated with mTORi	Proposed Strategies to Prevent AEs
Dyslipidemia: hypercholesterolemia, hypertriglyceridemia	45%, 50% [64]	Reduction of mTORi exposure, administration of statins and other dyslipidemia drugs
Hyperglycemia	17% [101]	Lifestyle modifications, pharmacological management of diabetes mellitus
Proteinuria	3% [78]	Constant monitoring of proteinuria when >800 mg/d, administration of ACEi or ARBs
Wound healing complications	11% [18,21]	Delayed introduction of mTORi at 4–6 weeks after LT, correction of the risk factors for WHC, administration of the minimum effective doses
Hematologic side effects: anemia, neutropenia, thrombocytopenia	14%, 11%, 9% [102]	Adjustment of mTORi exposure
Mucosal and integumental adverse effects: oral ulcers, dermatitis, leg edema	24%, 25%, 57% [103]	Oral hygiene, avoidance of irritant food and beverages, topical treatment of oral ulcers, adjustment of mTORi exposure

## Data Availability

Data is contained within the article.
